# Reg-1α Promotes Differentiation of Cortical Progenitors via Its N-Terminal Active Domain

**DOI:** 10.3389/fcell.2020.00681

**Published:** 2020-08-13

**Authors:** Marjorie Varilh, Isabelle Acquatella-Tran Van Ba, Michelle Silhol, Francisco Nieto-Lopez, Mireille Moussaed, Marie-Christine Lebart, Paola Bovolenta, Jean-Michel Verdier, Mireille Rossel, Anne Marcilhac, Françoise Trousse

**Affiliations:** ^1^MMDN, Univ Montpellier, EPHE, INSERM, Montpellier, France; ^2^PSL Research University, Paris, France; ^3^Centro de Biología Molecular Severo Ochoa, Consejo Superior de Investigaciones Científicas – Universidad Autónoma de Madrid and CIBER de Enfermedades Raras, Madrid, Spain

**Keywords:** neural progenitors, Reg-1α, Extl3, axon elongation, differentiation, GSK-3β

## Abstract

Reg-1α belongs to the Reg family of small, secreted proteins expressed in both pancreas and nervous system. Reg-1α is composed of two domains, an insoluble C-type lectin domain and a short soluble N-terminal peptide, which is released from the molecule upon proteolytic N-terminal processing, although the biological significance of this proteolysis remains unclear. We have previously shown that binding of Reg-1α to its receptor Extl3 stimulates axonal outgrowth. *Reg-1α* and *Extl3* genes are expressed in the developing cortex but their expression decreases in adulthood, pointing to a possible function of this signaling system at the early developmental stages. Here, we demonstrate that recombinant Reg-1α increases migration and differentiation of cultured embryonic rat telencephalic progenitors via the activation of GSK-3β activity. *In vivo* overexpression of Reg-1α by *in utero* electroporation, has a similar effect, favoring premature differentiation of cortical progenitors. Notably, the N-terminal soluble domain, but not the C-type lectin domain, is largely responsible for Reg-1α effects on cortical neuronal differentiation. We thus conclude that Reg-1α via its proteolytically generated N-terminal domain is required for basic development processes.

## Introduction

Reg-1α (Regenerating islet-derived-1α gene) belongs to the large family of Reg proteins (Watanabe et al., 1990; Laurine et al., 2005), the first member of which was discovered over four decades ago. Reg-1α is expressed in various organs and is specifically involved in cell proliferation and differentiation of cells of the digestive system with a paracrine/autocrine mechanism (Zhang et al., 2003; Miyaoka et al., 2004). Reg-1α is also expressed in the central nervous system (CNS) in a developmentally regulated manner. Reg-1α is strongly expressed in fetal and postnatal brain but very weakly in the normal adult brain (de la Monte et al., 1990), in which its expression has been associated with neuronal sprouting and regeneration (de la Monte et al., 1990; Xu et al., 1995). There is increasing evidence showing Reg-1α re-expression in pathologies, especially associated with the neuroinflammation present in the Alzheimer’s disease pathology (AD) (de la Monte et al., 1990; Duplan et al., 2001; Grégoire et al., 2001; Laurine et al., 2005). In this context, we have previously uncovered that Reg-1α is upregulated at very early stage of AD and in an aging model, the primate mouse lemur (Marchal et al., 2012). In human AD brains, Reg-1α forms deposits, which are proportional to neurofibrillary tangles’ distribution, suggesting a Reg-1α relationship with tangles’ evolution. This elevated Reg-1α expression has been proposed to reflect a widespread aberrant neuritic sprouting associated with synaptic alterations and dementia (de la Monte et al., 1996; Laurine et al., 2003; Marchal et al., 2012). Reg-1α colocalizes with hyperphosphorylated Tau in neurofibrillary tangles from AD and progressive supranuclear palsy (PSP) brains. Furthermore, in cellular tauopathy models, Reg-1α increases Tau phosphorylation via AKT/GSK-3β pathway leading to morphological abnormalities of neurons and axonal transport defects (Moussaed et al., 2018).

Reg-1α is highly susceptible to proteolysis (Cerini et al., 1999) and its cleavage generates a soluble N-terminal undecapeptide and a C-type-lectin terminal portion of 133 amino acids that precipitates and forms protease-K-resistant fibrils at physiological pH (Cerini et al., 1999; Milhiet et al., 2010). The *in vitro* cleavage of Reg-1α leads to the formation of quadruple-helical fibrils, composed of C-type lectin domain multimers, whereas the presence of an uncleaved N-terminal undecapeptide blocks dimers’ assembly into fibrils of high order (Cerini et al., 1999). Despite this information, the physiological relevance of Reg-1α proteolytic products in the mammalian CNS is unknown.

Reg-1α binds to the Extl3 receptor, initially isolated from a rat pancreatic islet (Kobayashi et al., 2000). Extl3 belongs to the Exostosin tumor-like (Ext) family and contains a membrane-spanning and a Reg-1α binding domain. *Extl3* contributes to brain development with a developmentally regulated expression (Osman et al., 2004). *Extl3* mRNA is strongly expressed in the developing rodent brain and spinal cord (Osman et al., 2003, 2004) with a distribution that suggests involvement in neural precursor cell proliferation, migration and neuron generation. In agreement with this notion, secreted Reg-1α stimulates neurite outgrowth via Extl3 *in vitro* (Acquatella-Tran Van Ba et al., 2012) but the precise role of Reg-1α during mammalian brain development is still poorly explored.

On the basis of this information and on the observation that Reg-1α acts as differentiation factor for different cell types (Jing et al., 2010; Parikh et al., 2012; Chen et al., 2019), here we have investigated the potential function of Reg-1α on migration and differentiation of neural precursor cells. Using neurosphere cultures and *in utero* electroporation, we show that Reg-1α increases migration and differentiation of rodent neural and cortical precursors through the GSK-3β/β-catenin pathway and promotes the *in vivo* differentiation of cortical precursors, thereby modifying their migration. Notably, the Reg-1α N-terminal undecapeptide is necessary and sufficient to mimic this function. Thus, these data demonstrate a novel and GSK-3β mediated function of Reg-1α and provide the first demonstration of a physiological role for its N-terminal cleavage product during the neurodevelopmental processes.

## Materials and Methods

### Animals

Sprague-Dawley rats (Depré, St Doulchard, France) and C57BL/6J mice (Elevage Janvier, Le Genest Saint Isle) were housed and cared for, following the animal welfare guidelines established by the INSERM and the Montpellier University. All animal procedures were performed in strict adherence to the European Union directive 2010/63 and the ARRIVE guidelines (Kilkenny et al., 2010) upon CEEA-LR-12031 authorization.

### RNA Extraction and cDNA Synthesis

Total RNA was extracted from neuro-spheres and brain using the NucleoSpin^®^ 8/96 RNA kit (Macherey-Nagel, Germany) according to the manufacturer’s instructions. RNA concentration and purity were evaluated with the Agilent RNA 6000 Nano Kit (Agilent Technologies, Waldbronn, Germany). Only RNAs with an RNA Integrity Number (RIN) > 8 were used. cDNA was synthesized in a 20 μl reaction mixture. One μg of total RNA was mixed with 1 μl random primer hexamer (0.5 μg/μl) (Amersham, Orsay, France) and incubated at 70°C for 10 min. After cooling on ice, the solution was mixed with 4 μl of 5X first strand buffer, 2 μl of 0.1 M DTT, 1 μl each of dATP, dTTP, dCTP, dGTP (each 10 mM) and 1 μl MuMLV reverse transcriptase (200 U; Promega, Charbonnieres, France) and incubated at 37°C for 60 min and then at 70°C for 15 min. Parallel reactions for each RNA sample were run in the absence of MuMLV to assess the degree of genomic DNA contamination. Obtained amplicons were stored at −20°C until use.

### DNA Constructs

Deleted versions of Reg-1α were obtained with a PCR-based mutagenesis protocol. The coding sequence of human Reg-1α (GenBank Accession Number 002909.5; OriGene Technologies) was subcloned in the pCAGGS/ES vector (Addgene). The CDS of human Reg-1α in pCAGGS/ES was used as template to generate the deleted versions Reg-1α^Δ23–33^ lacking 11 amino acids between the signal peptide and the proteolytic site (33 AA), and Reg-1α^Δ34–166^, lacking the C-terminal domain of the protein.

Reg-1α^Δ^ ^23–33^ was generated combining PCR reactions. The upstream region of human Reg-1α encompassing the region for amino acid 1-22 was amplified by the primer 5′-GGGGTACCATGGCTCAGGCCAGCTCA-3′ including translation initiation start (ATG) and KpnI site and the primer 5′-GCCTTCTGGGCAGCTGATGCCTTGGCTCTGAGACAG-3′. The downstream region of the protein (from amino acid 34) was amplified using the 5′-CTGTCTCAGAGCCA AGGCATAGCTGCCCAGAAGGC-3′ and the 5′-GGGAGC TCTAGTTTTTGAACTTGCAGAC-3′ primers including translation initiation stop and SacI site. The two PCR products were mixed and amplified in a third PCR reaction using primers 5′-GGGGTACCATGGCTCAGGCCAGCTCA-3′ and 5′-GGGAGCTCTAGTTTTTGAACTTGCAGAC-3′. The final PCR product encoding CDS human Reg-1α^Δ^ ^23–33^ was cloned in the pCAGGS/ES vector. Human Reg-1α^Δ^ ^34–166^ was constructed by PCR amplification using the forward 5′-GGGGTACCATGGCTCAGGCCAGCTCA-3′ and reverse 5′-GGGAGCTCTA GTTTTTGAACTTGCAGAC-3′ primers, including translation initiation stop and SacI site. Full length mouse Reg-1α cDNA (BC-028761, openBIOSYSTEMS) was sub-cloned into the pCAGGS/ES vector. All plasmids were sequenced and contained the signal peptide that allows protein secretion. The pCAG-GFP plasmid, allowing expression of green fluorescent protein (GFP), was used as a positive transfection control.

### Quantitative Real-Time PCR

Real-time PCR was performed using a LightCycler^®^ 480 Instrument (Roche Diagnostics, Mannheim, Germany) according to the manufacturer’s instructions. PCR reactions were performed in a final volume of 6 μl with 1 × LightCycler 480 SYBR Green I Master mix, 1 μM of each primer and 1/12.5 diluted RT mixture as PCR template (or water as negative control). The amplification conditions were: (i) cDNA denaturation (1 cycle: 95°C for 10 min); (ii) amplification (45 cycles: denaturation at 95°C for 10 s, annealing at 60°C for 10 s, elongation at 72°C for 10 s); (iii) melting curve analysis (1 cycle from 65 to 95°C for 60 s); (iv) cooling (1 cycle: 40°C for 60 s). Real-time detection of the SYBR Green I fluorescence intensity, indicating the amount of PCR product formed, was measured at the end of each elongation phase. To confirm the amplification specificity, PCR products from each primer pair were subjected to a melting curve analysis and then separated by agarose gel electrophoresis. The amounts of target genes were normalized relative to *cyclophilin* used as reference gene in the corresponding samples. Primers previously described (Jing et al., 2010) were used with some modifications. The sequences of the primers specific for the examined genes and the predicted product sizes are shown in Table 1.

**TABLE 1:** Primers and expected sizes of the corresponding PCR products.

Gene	Forward primer	Reverse primer	Size (bp)
*Cyclophilin*	5**′**-ataatggcactggtggcaag-3′	5**′**-catgccttctttcaccttcc-3′	199
Reg-1α	5′-caggctacctggtgtcagt-3′	5′-cacagtagccacgattggaa-3′	219
*Extl3*	5′-tgtgaggatatcgccatgaa-3′	5′-tgctcatcgtctcctcagaa-3′	313

### Western Blot Analysis

Frontal cortex was dissected from male rats at different ages and disrupted with a Dounce homogenizer in cold lysis buffer (20 mM Tris, pH 7.5, 100 mM NaCl, 0.1% Triton, 1 mM EGTA) with Complete Mini protease inhibitor mixture (Roche Applied Science). Protein concentration was determined with the BCA assay (Pierce), and 20 μg of proteins were loaded in 12.5% SDS-PAGE. For protein/peptide detection, nitrocellulose membranes (0.22 μm, Bio-Rad) were incubated with primary antibodies at 4°C overnight and with secondary antibodies at RT for 2 h. Immunolabeling was revealed by chemiluminescence reaction using the ECL Western blot detection reagents (Luminata crescendo western HRP substrate, Millipore) and measured using the Licor detection system. Primary antibodies were: rabbit anti-Reg-1α [1:500 (Duplan et al., 2001)], goat anti-EXTL3 (1:1000, #AF2635, R&D Systems), rabbit anti-p-GSK-3β Ser^9^ (1:1000,#9336S, Cell Signaling); mouse anti-GSK-3β (1:5000, # 610201, BD Transduction laboratory), mouse anti-p-GSK-3β Tyr^216^ (1:1000, # 612313, BD Transduction laboratory) anti-β-catenin (1:5000,#610153, BD Transduction laboratory) and mouse anti-β-actin (1:5000,#A5441, Sigma-Aldrich). Generation and specificity of the anti-Reg-1α rabbit polyclonal antibody has been previously reported (Duplan et al., 2001; Laurine et al., 2003; Acquatella-Tran Van Ba et al., 2012; Moussaed et al., 2018).

### Neurosphere of NPCs Culture

Neural Precursor Cells were obtained from telencephalon of E13.5 Sprague-Dawley rat embryos. Cells were dissociated enzymatically by incubation in a solution of trypsin and 0.05% EDTA, and then mechanically by repeated pipetting. After filtration through a 70 μm cell strainer, cells were resuspended in proliferation medium constituted by DMEM/F12 medium supplemented with 1% N2 (Invitrogen), 1% antibiotics (penicillin/streptomycin), 2 mM glutamine, 20 ng/mL bFGF and 10 ng/mL EGF (Invitrogen). Cells were grown in non-adherent culture conditions in flasks coated with polyhydroxyethyl metacrylate (Sigma) at 37°C and 84% hygrometry. Small floating cell clones were observed from day 2 after plating and formed neurospheres by clonal expansion (Armando et al., 2007). Neurospheres were passaged every week. At passage 3 (P3), neurospheres were equally divided among the different experiments, centrifuged at 300 *g* for 5 min and each pellet was resuspended in the appropriate medium (with or without growth factors) and plated on an adherent support. Neurospheres were seeded on 10 μg/mL poly-D-lysine-coated 12 mm glass coverslips in 24-wells dishes for immunocytochemical studies, or directly on poly-D-lysine-coated 35 mm dishes or 6-wells plates for RNA extraction, western blot and FACS analysis. Cells were cultured at 37°C in humidified air with 5% CO_2_ for 48 h.

### Subcellular Fractionation and Immunoblotting

NSCs were washed twice with PBS and re-suspended in lysis buffer (10 mM HEPES, pH 7.5, 10 mM NaCl, 1 mM EDTA, 1 mM DTT) with complete mini protease inhibitor cocktail (Roche Biomedicals). Cells were allowed to swell on ice for 15 min and then they were homogenized with a Dounce homogenizer. Nuclei integrity was verified under a light microscope and homogenates were layered on 40% sucrose. The nuclei were collected by centrifugation at 2000 rpm for 15 min and the nuclear pellets were re-suspended in 100 μl of the same buffer. The supernatants were then centrifuged at 13,000 rpm at 4°C for 30 min to separate the cytosol (supernatant) and membrane (pellet) fractions. Protein concentration was determined with the BCA assay (Pierce). Cytosolic and nuclear fractions (20 μg) were loaded on 12.5% SDS-polyacrylamide gels (SDS-PAGE).

### *In utero* Electroporation and 5-Bromo-2-Deoxyuridine Labeling

Pregnant mice were ethically anesthetized with isoflurane combined with oxygen (TEM SEGA). The mice were coinjected with pCAG-GFP and pCAG expressing different constructs of Reg-1α (1.5 μg/μl) in a 1:3 ratio, were mixed with 0.5 μl Fast Green (0.03%, Sigma) and injected (2 μl) into the lateral ventricle of E14.5 embryonic brains using a pulled glass micropipette. Electrode pair (5 mm in diameter; CUY650-P5, NEPA Gene) attached to an ECM 830 electroporator (BTX Harvard Apparatus) transmitted 5 squares 40 V electric pulses for 50 ms at 950 ms intervals through the uterine wall. Each condition was applied to at least three independent litters, targeting the same dorso-lateral region of the neocortex. For 5-bromo-2-deoxyuridine labeling (BrdU, Sigma), electroporated pregnant dams were intraperitoneally (i.p) injected with BrdU (100 μg/g) 1 day after electroporation and sacrificed 24 h later. The sections were stained for rabbit anti-T-box brain 1 (Tbr1) to delineate the cortical plate. The percentage of targeted cells was determined by dividing the number of GFP positive cells in each layer (cortical plate and intermediate zone/ventricular zone) by the total number of GFP-positive cells in the entire section.

### Tissue Processing and Immunostaining

Neural progenitor cultures were fixed in formaldehyde/PBS for 30 min followed by three washes in PBS. Cells were blocked and permeabilized in PBS supplemented with 2% BSA and 0.1% Triton and then incubated at 4°C with primary antibodies. Animals were anesthetized with ketamine (Imalgen 1000) and xylazine (Rompun 2%) (75 and 25 mg/kg IP, respectively) and perfused with 40 ml of phosphate buffered saline (PBS) and 40 ml of 4% paraformaldehyde in 0.1 M PBS. Adult brains were post-fixed for 48 h in 4% paraformaldehyde and rinsed in PBS. Embryonic brains were post-fixed for 4 h at 4°C. Samples were cryoprotected in 30% sucrose solution and embedded in O.C.T. compound (Sakura). Embedded tissues were sectioned on a cryostat (14 μm thickness) in the coronal plane. For analyses of *in utero* electroporation, brains were dissected after intracardiac perfusion of the embryos with antigenfix solution (Microm Microtech), 2 or 3 days following electroporation. Brains were post-fixed for 4 h at 4°C, rinsed in 0.1M phosphate buffer (pH 7.4, Sigma) and immersed in a 10% sucrose solution diluted in phosphate buffer overnight at 4°C for cryoprotection. Brains were sectioned at 20 μm in the coronal plane. The following primary antibodies were used: mouse anti-Nestin (1:200, #4760, Cell Signaling), mouse anti-βIII Tubulin (1:400, #T8660, Sigma), mouse-NeuN (1:200, clone A60, #MAB377, Millipore), rabbit anti-CalB (1:1000, #CN-38a, Swant), rat anti-Brdu (1:250, #ab6326, Abcam) rabbit anti-T-box brain 1 (Tbr1, 1:300, #AB10554, Millipore), rabbit anti-Ki67 (1:100, #ab16667, Abcam) and rabbit anti-Reg-1α (1:500, Duplan et al., 2001). Electroporated cells were identified by GFP expression. For BrdU labeling, sections were incubated in 2N hydrochloric acid for 20 min at RT followed by neutralization with 100 mM sodium borate (Sigma), pH 8.9, for 15 min before the permeabilization step. Secondary antibodies directed against rabbit or mouse and rat IgGs conjugated to Alexa Fluor 488 (1:1000, Invitrogen) and Cy3 or Cy5 (1:400, Jackson Immuno-Research Laboratories) were incubated 2 hours at room temperature and then the nuclei were counterstained with 4′, 6′-diamino-2-phenylindole (DAPI, Molecular Probes). Sections were imaged with confocal microscopy with a Leica TCS4D confocal laser-scanning microscope (Leica Microsystems, Heidelberg, Germany). Images were processed using Adobe Photoshop (Adobe Systems Inc., Mountain View, CA, United States).

### Measurement of Migration and Differentiation

To quantify neural precursor cells migration and differentiation, we measured the shape of 10 neurospheres, the distance covered by migrating cells and βIII-Tubulin expression level in cells outside neurospheres. The perimeter of each neurosphere after DAPI staining was calculated with ImageJ software. The number of migrating cells was defined as the total number of cells outside each neurosphere, determined using the cell counter plug-in. The distance between the border of each neurosphere and the 10 furthest cells was evaluated and taken as an estimate of the migratory capacity of the cells. Additional quantifications using the NeuronJ plug-in included: (i) the intensity of βIII-Tubulin staining around each neurosphere and (ii) the longest neurite calculated by sampling 10 cells, the soma of which was located at the edge of the neurosphere. Images were obtained by imaging neurospheres using a Zeiss Axiovert microscope and a MRm2 camera system (Supplementary Figure 1).

### FACS Analysis

Neural precursor cells were washed with PBS and then incubated with a trypsin/0.05% EDTA solution at 37°C for 5 min. Dissociated cells were centrifuged at 300 *g* for 5 min (and also after each of the following steps). Pellets were washed once with PBS and fixed in formaldehyde/PBS solution for 30 min followed by another wash in PBS. Cells were blocked and permeabilized in PBS supplemented with 2% BSA and 0.1% Triton and then incubated at 4°C with mouse anti-βIII-Tubulin or anti-Nestin antibodies in the same buffer overnight. The next day, cells were incubated with a secondary antibody directed against mouse IgGs coupled to Alexa Fluor 488 (Invitrogen). Cells were analyzed using a BD FACSCanto^TM^ cell analyzer (BD Biosciences) with the running FACS Diva v6.1.3 software for acquisition. Data were processed with the FlowJo software.

### Primary Hippocampal Neuronal Cultures

Hippocampal neurons were isolated from 17.5-days-old embryos of timed pregnant Sprague-Dawley rat (CE Depré, Saint Doulchard, France) as previously described (Acquatella-Tran Van Ba et al., 2012). In brief, the hippocampi from embryonic brains were dissected in PBS supplemented with 6% glucose, 10 units/ml penicillin/streptomycin and digested with 0.05% trypsin-EDTA. The cell suspensions were plated on 18-mm glass coverslips (25.10^4^ cells/well for transfection), or 12-mm glass coverslips (5.10^4^ cells/well for treatment). Cells were re-suspended in Neurobasal medium with 0.5% B27, 50 μm L-Glutamine, 0.25% Glutamax, 1% penicillin/streptomycin and 10% FBS. 5 × 10^5^ freshly dissociated hippocampal cells were transfected using 4D-Nucleofector^R^ System Nucleofector^TM^ (Amaxa Biosystems, Cologne, Germany), using the program N/Rat Hipp-cortical HV, pulse code CU 133. The transfected cells were then seeded on glass coverslips in 24-well plates coated with 10 μg/ml poly-D-lysine to study neurite elongation. Cells were then cultured at 37°C for 48 h in humidified air with 5% of CO_2._ For the analysis of neuronal morphology, *a*xonal like processes (Tau-positive/MAP2-negative) from isolated transfected and non-transfected neurons were visualized and photographed using a Axioimager Leica fluorescent microscope equipped with an Axiovision LE 4.4 software. The length of the axons was measured using the NeuronJ software (ImageJ), tracing β3 Tubulin-positive fibers.

### Statistical Analysis

All bar graphs are plotted as mean ± SEM. The values obtained in electroporation conditions were compared using GraphPad InStat (San Diego, CA, United States). Significance of the percentage of GFP-positive cells in the CP and in IZ/VZ for each condition was determined using Mann Whitney test. One-way ANOVA of cell cycle exit and neurite outgrowth revealed significant differences among experimental groups, multiple comparisons among experimental groups were performed using Dunnett’s or Bonferroni’s *post hoc* test.

## Results

### Age-Dependent Expression of Reg-1α Protein in the Cortex Tissue

**FIGURE 1:**
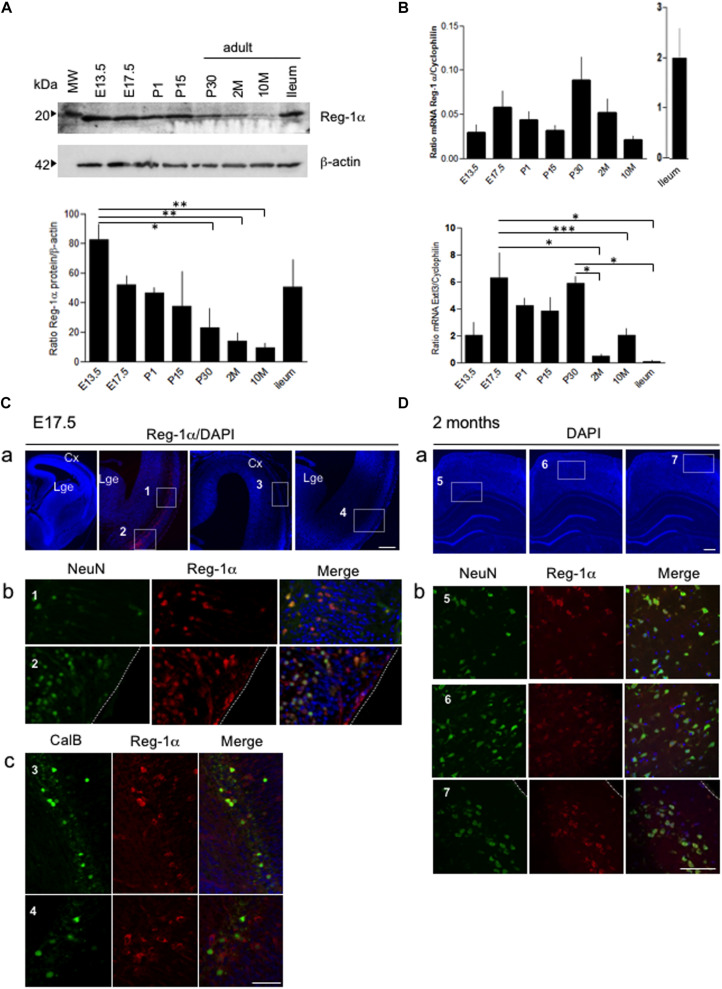
Age-dependent expression of Reg-1α protein and Extl***3*** in brain tissues. **(A)** Levels of Reg-1α protein were determined using western blotting of cortical tissue extracts prepared from rats of the following ages: E13.5 and E17.5 (*n* > 10 per group), P1, P15, and P30 (*n* > 4 per group) and 2, 10 months-old (2M, 10M) (*n* > 3 per group). Extract of ileum from 2 months-old animals was used as positive control. Relative Reg-1α protein levels were normalized with β-actin values and plotted according to the age group. **(B)** qPCR analysis of Reg-1α and *Extl3* gene expression in brain homogenates from the same developmental stages used above. Values were normalized to *cyclophilin* expression. **(A,B)** Bars represent the mean from independent embryos per group. One-way ANOVA followed by Bonferroni’s multiple comparison test. Statistical significance: *p < 0.05, **p < 0.01, ***p < 0.001. **(C)** Coronal cryosections of E17.5 cortex immunostained for Reg-1α, NeuN and Calbindin. Note that Reg-1α colocalizes with NeuN in the dorso-lateral (**a**, boxed area 1) and ventral cortex (**a**, boxed area 2) and with Calbindin in interneurons of the lateral and piriform cortex (**a**, boxed areas 3–4). High magnification pictures in **(b,c)** correspond to the boxed areas in **(a)**. **(D)** Double immunostaining for Reg-1α/NeuN in the parietal cortex of 2 months-old rats shows double-positive cells scattered through all cortical layers (**a**, boxed areas 5-6-7). High magnification of the boxed areas are illustrated in **(b)**. Cx, cortex; Lge, Lateral ganglionic eminence. Scale bars, 80 μm.

We have previously demonstrated that secreted Reg-1α stimulates axon outgrowth from cultured neurons via Extl3 (Acquatella-Tran Van Ba et al., 2012), suggesting a possible physiological role during brain development. In developing brain, Reg-1α is known to be expressed (de la Monte et al., 1990), but its exact distribution is still poorly explored. To address whether Reg-1α is involved in cortical neurogenesis, we thus first investigated its expression pattern in the developing rat cortex. Western blot analysis of brain extracts at different developmental stages (from E13.5 to adulthood) revealed the presence of a specific 18 kDa band (Figure 1A), corresponding to the reported Reg-1α MW found in the adult small intestine (ileum) (Namikawa et al., 2005) and here used as a control. Quantification of identified band revealed a progressive developmental decrease, so that 1 month old brains had threefold less protein that E13.5 brains, with further decrease with aging (Figure 1A). However, the quantitative RT-PCR of Reg-1α transcript expression normalized to the levels of *cyclophilin* mRNA did not show any significant difference between the different stages and did not corroborate with expression kinetics (Figure 1B compare to Figure 1A). Quantitative RT-PCR of *Extl3* expression, normalized to the levels of *cyclophilin* mRNA, showed a high expression at early stages (between E17.5 and P30) of cortical development with decreased expression in the adult tissue (Figure 1B).

Double immunostaining for Reg-1α and either the pan-neuronal NeuN or the immature GABAergic interneuron Calbindin (CalB) markers identified positive neuronal subtypes in the cortex at E17.5 (Figure 1C). Reg-1α colocalized with NeuN in the cortical plate and piriform cortex (respectively box1 and 2 in Figure 1Ca; higher magnifications in Figure 1Cb), whereas Reg-1α and Calbindin were co-expressed in a restricted interneuron subpopulation concentrated in superficial layer (Figure 1C, boxes 3–4; higher magnifications in Figure 1Cc). Reg-1α was still detectable in NeuN positive cells in all layers of the adult parietal cortex (Figure 1D, boxes 5–7; higher magnifications in Figure 1Db).

### Characterization of Neurospheres

**FIGURE 2:**
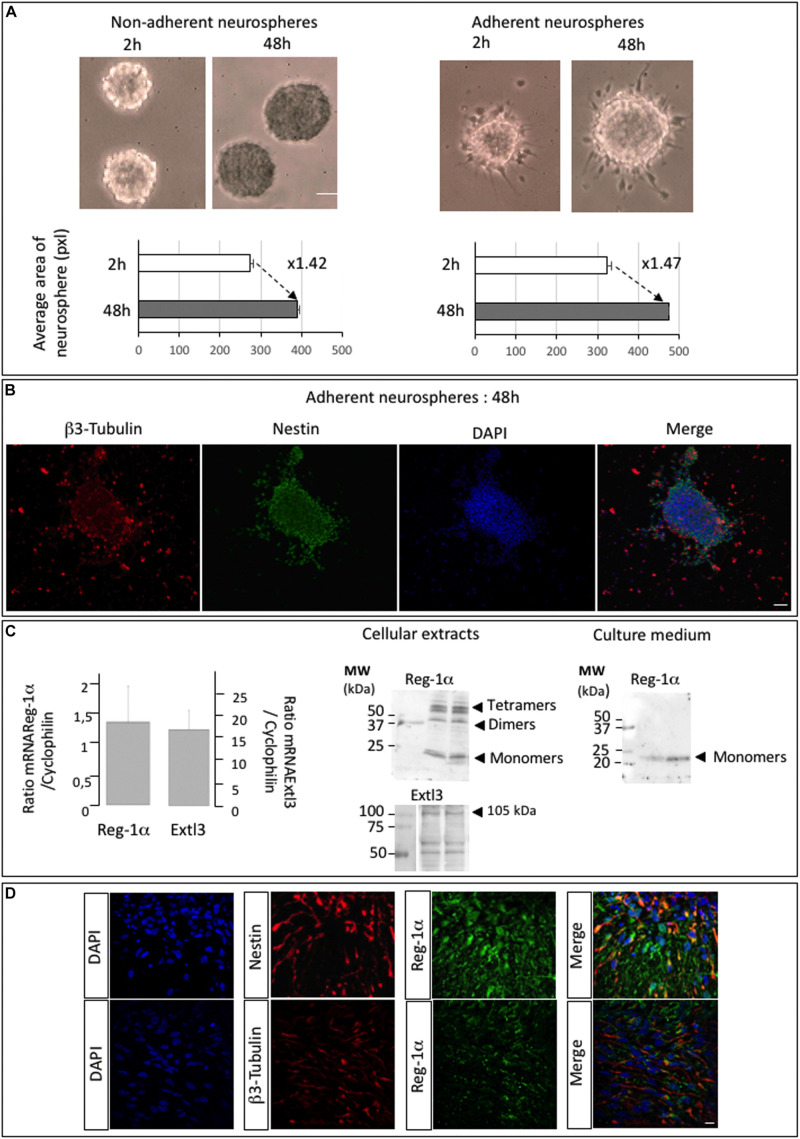
Neurospheres of telencephalic embryonic precursor cells express Reg-1α and Extl3. **(A)** Bright field images of neurospheres at 2 and 48 h of culture on non-adherent and adherent substrate. The graph shows the quantification of neurosphere size (pixel areas). Scale bar, 100 μm. **(B)** Adherent neurospheres after 48 h of culture immunostained for β3-Tubulin and Nestin. β3-Tubulin-positive differentiated cells are predominant outside the neurosphere whereas Nestin-positive cells localize inside and at the edge of the neurosphere. Scale bar, 50 μm. **(C)** RT-qPCR analysis of *Reg-1*α and *Extl3* mRNA expression in neurospheres grown for 48 h on an adherent substrate (left panel). Values were normalized to those of *cyclophilin* expression. Western blots of cellular extracts and culture medium from neurospheres grown on an adherent substrate for 48 h (right panel). Reg-1α and Extl3 are expressed in the cells. Reg-1α is secreted in the medium. **(D)** Immunofluorescence analysis of Reg-1α, Nestin, and β3-Tubulin expression in neurospheres. Merged images show that Reg-1α localizes to Nestin-positive progenitors and in β3-Tubulin-positive post-mitotic neurons in both cell bodies and neuritic extensions. Scale bar, 10 μM.

The abundant expression of Reg-1α at early stages (E13.5) of telencephalic development suggested a role in migration and differentiation of neural progenitors. To assess this possibility, we used neurosphere (NS) cultures from rat embryonic telencephalon and tested their adhesion and proliferation properties. We plated free-floating NS after 3 passages on plates with or without poly-D-lysine coating and cultured them in proliferation medium for 2 and 48 h. Measure of the NS size showed no significative differences between non-adherent and adherent neurospheres (Figure 2A) and in both conditions the NS size increased about 1.5 times between 2 and 48 h. We thus selected to use adherent neurospheres grown in proliferation medium for 48 h to be able to measure their differentiation and migration processes (Supplementary Figure 1). NS contained cells at various stages of differentiation including from progenitors to post-mitotic neurons as illustrated by Nestin and β3-Tubulin immunostaining. Nestin-positive cells were localized inside and at the periphery of the NS whereas β3-Tubulin positive neurons were found mostly on the outside (Figure 2B), indicating a gradient of differentiation toward the periphery.

Notably, quantitative RT-PCR and western blot analysis of the NS detected mRNA and protein expression for both *Reg-1α* and *Extl3* (Figure 2C). Reg-1α was also expressed in culture medium of NS (Figure 2C, right panel) indicating that the protein is secreted by neural cells, as previously shown for hippocampal neurons (Acquatella-Tran Van Ba et al., 2012). As NS contain cells at various stages of differentiation, including Nestin-positive proliferating progenitors and β3-Tubulin positive post-mitotic neurons, we next asked if Reg-1α is present in both populations. Confocal microscopy analysis showed that this was the case (Figure 2D).

### Reg-1α Stimulates Neural Precursor Cell Migration and Differentiation

**FIGURE 3:**
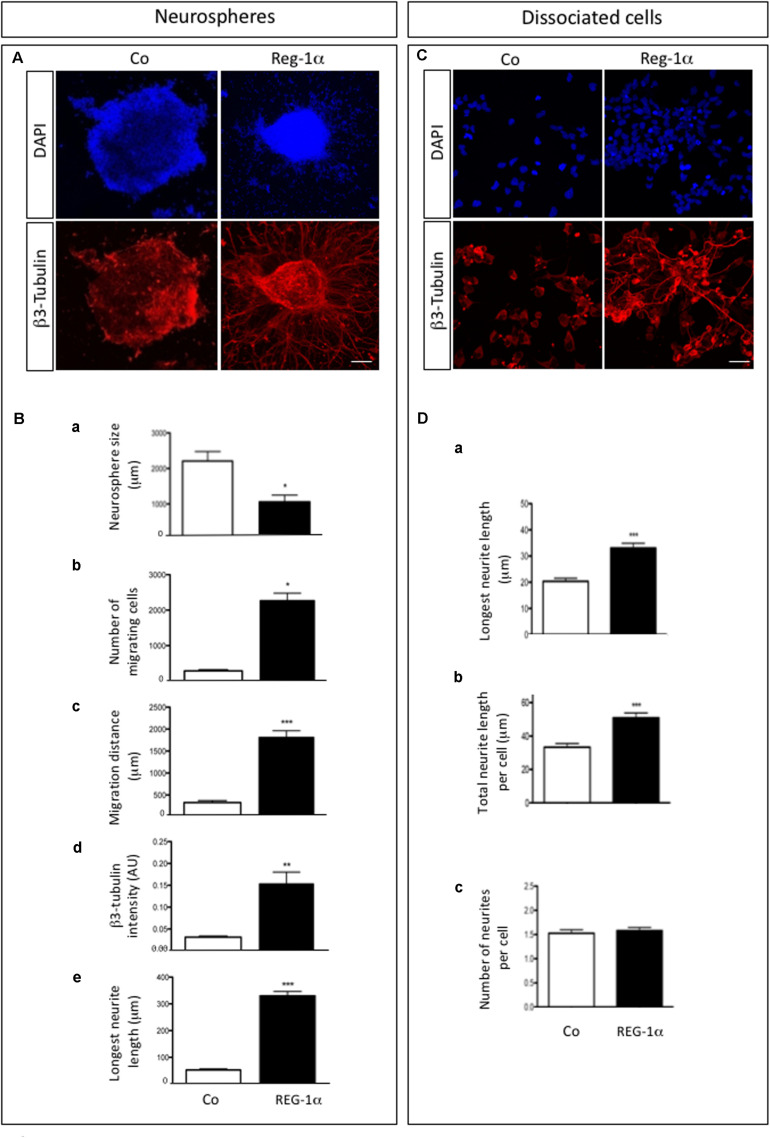
Reg-1α induces differentiation, migration and processes elongation growth of telencephalic precursor cells. **(A)** Neurospheres grown on an adherent substrate in proliferation medium in absence (Co) or presence of 10^– 7^ M recombinant Reg-1α for 48 h (Reg-1α). DAPI and β3-Tubulin immunostaining shows the effect of Reg-1α. Scale bar, 50 μM. **(B)** Quantification of neurosphere size **(a)**, number of migrated cells **(b)**, cell distance from the neurosphere edge **(c)**, β3-Tubulin staining intensity outside the neurosphere **(d)** and length of the longest neurite in β3-Tubulin-positive cells **(e)** in the two experimental conditions. Reg-1α treatment promotes cell differentiation of neurospheres. **(C)** Dissociated neural precursor cells immunostained for β3-Tubulin in presence or absence of 10^– 7^M recombinant Reg-1α. Scale bar, 25 μM. **(D)** Reg-1α significantly increases the length of the longest neurite **(a)** and the total neuritic length per cell **(b)** but not the number of neurites per cell **(c)**. Results represent three independent experiments Student’s *t*-test (mean ± SEM) *p < 0.05; **p < 0.01; ***p < 0.001.

We next asked whether Reg-1α could regulate neural precursor migration and differentiation. To this end, we analyzed adherent NS grown in proliferation medium in the presence of 10^–7^ M recombinant Reg-1α for 48 h (Figure 3), which are conditions previously shown to increase neuritic outgrowth (Acquatella-Tran Van Ba et al., 2012). β3-Tubulin and DAPI staining showed a significant decrease in NS size and an increase in the number of differentiated cells present outside of the NS upon Reg-1α (Figures 3A,B, panels a–c, black bars *vs.* white bars). Reg-1α also increased the intensity of β3-Tubulin staining and neurite length compared to control, suggesting that Reg-1α promotes neural precursor cell differentiation (Figures 3Bd,e). We next switch the NS culture medium to a previously described differentiation medium (Armando et al., 2007) and show that the results obtained with recombinant Reg-1α treatment are comparable with those obtained with the differentiation medium (Supplementary Figure 2).

To assess if Reg-1α had a direct effect on neurite outgrowth, we tested the effects of Reg-1α on dissociated adherent cells derived from telencephalic NS (Figure 3C). β3-Tubulin staining showed that Reg-1α increases neuritic elongation: the total neuritic length and the length of the longest neurite were increased in treated vs. untreated cells (Figures 3Da,b) but no difference in the number of processes per cell was observed (Figure 3Dc). These results are in agreement with those previously reported for embryonic hippocampal neurons (Acquatella-Tran Van Ba et al., 2012).

**FIGURE 4:**
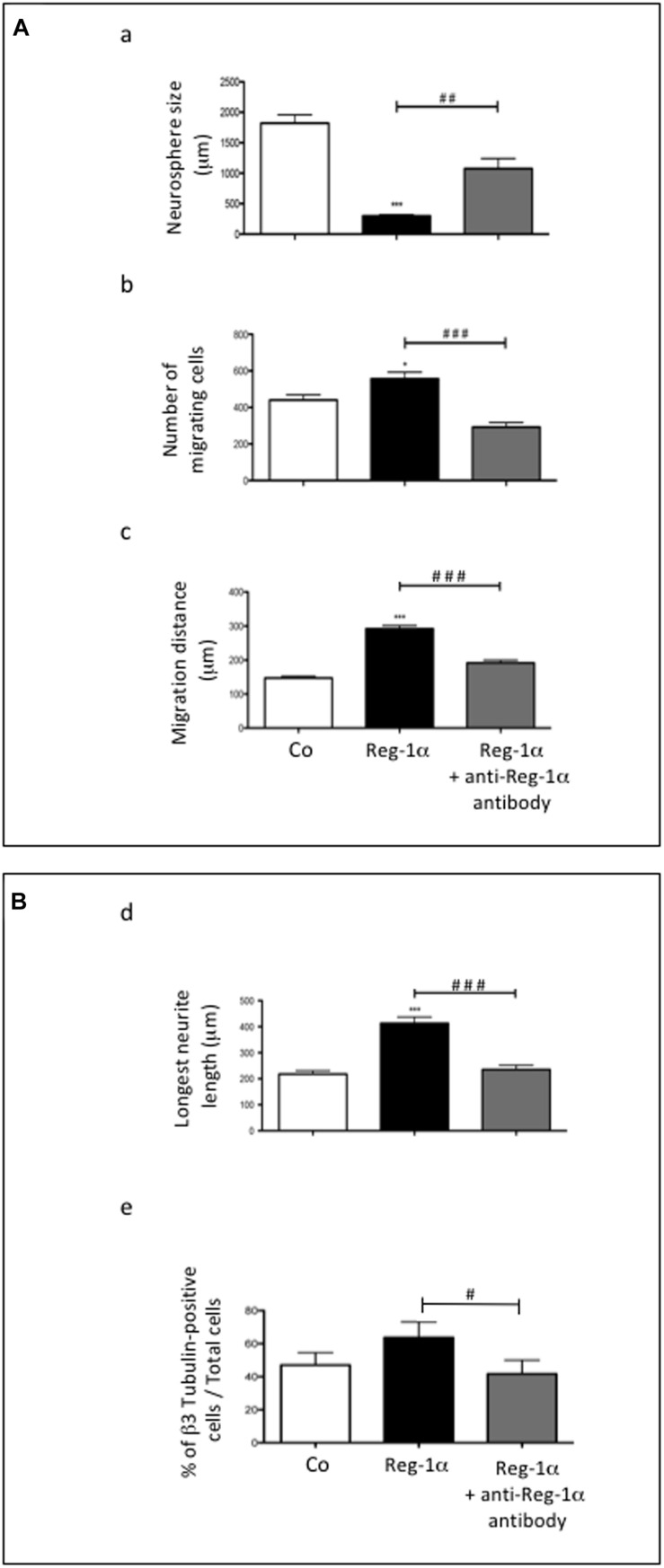
*Treatment with anti-Reg-1α antibodies neutralizes the effect of Reg-1α*. **(A,B)** Quantitative analysis of the neutralizing effect of anti-Reg-1α polyclonal antibody on the differentiative activity of Reg-1α on neurosphere cultures. Neurosphere size **(Aa)**, the number of migrating cells **(Ab)**, the distance from the neurosphere edge **(Ac)** and the length of the longest neurite **(Bd)** were measured. Flow cytometry analysis of the number of β3-Tubulin-positive cells in neurosphere grown in control conditions (proliferation medium alone, white bars), or in the presence of Reg-1α (10^–7^ M; black bars) or recombinant Reg-1α (10^–7^ M) + anti-Reg-1α antibody (10^–6^ M; gray bars) **(Be)**. The histograms show the quantification of three independent experiments (mean ± SEM). ^∗^*p* < 0.05; ^∗∗∗^*p* < 0.001 relative to control and ##*p* < 0.01; ###*p* < 0.001 relative to Reg-1α treated cells based on the Student’s *t*-test. #*p* ¡ 0.05.

To confirm the specificity of Reg-1α effects on neural precursor migration and differentiation, we blocked the effect of Reg-1α with the addition to the culture medium of a previously described anti-Reg-1α polyclonal antibody (Acquatella-Tran Van Ba et al., 2012). The antibody partially inhibited Reg-1α effect on precursor cells migration (Figures 4Aa–c, gray bars *vs.* black bars) and differentiation (Figures 4Bd,e, gray bars *vs.* black bars) determined as above.

All in all these data demonstrate that Reg-1α specifically promotes migration and differentiation of neural precursor cells from the telencephalon.

### Cross-Talk Between Reg-1α and GSK3-β Pathway

**FIGURE 5:**
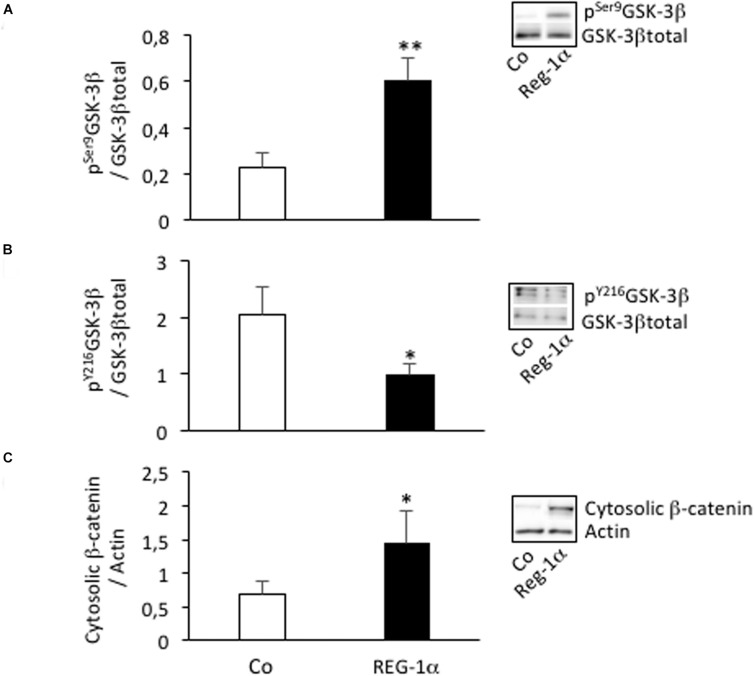
Reg-1α leads to changes in GSK-3β phosphorylation and β-catenin subcellular localization. Western blot assays of the levels of p^Ser9^GSK-3β **(A)**, p^Y216^GSK-3β **(B)**, total GSK-3β, and cytosolic β-catenin **(C)** from homogenates of neurospheres grown in proliferation medium alone (Co, white bars) or with the addition of Reg-1α (10^–7^ M; black bars). Note that Reg-1α enhances p^Ser9^GSK-3β/GSK-3β total and decreases p^Y216^GSK-3β/GSK-3β total ratios. Student’s *t*-test ^∗^*p* < 0.05; ^∗∗^*p* < 0.01.

Reg-1α regulates GSK-3β (Moussaed et al., 2018) and GSK-3β/β-catenin signaling is known to play a key role in stem cell migration and differentiation. We thus asked whether the observed Reg-1α effects were mediated via this pathway. Notably, incubation of adherent NS with Reg-1α significantly increased p^Ser9^GSK-3β and the cytosolic level of β-catenin (Figures 5A,C, black bars *vs.* white bars), both of which are indicators of pathway activation (Cohen and Frame, 2001). The levels of p^Y216^GSK-3β were instead decreased (Figure 5B, black bars *vs.* white bars), as expected given that this modification leads to an opposite effect on GSK-3β activity. These data suggest that Reg-1α acts, at least in part, on neural precursor cells migration and differentiation in a GSK-3β dependent manner.

All the results obtained *in vitro* on neural precursor cells led us to test *in vivo* the effect of Reg-1α on cortical development in mouse.

### Overexpression of Reg-1α in the Embryonic Mouse Neocortex

**FIGURE 6:**
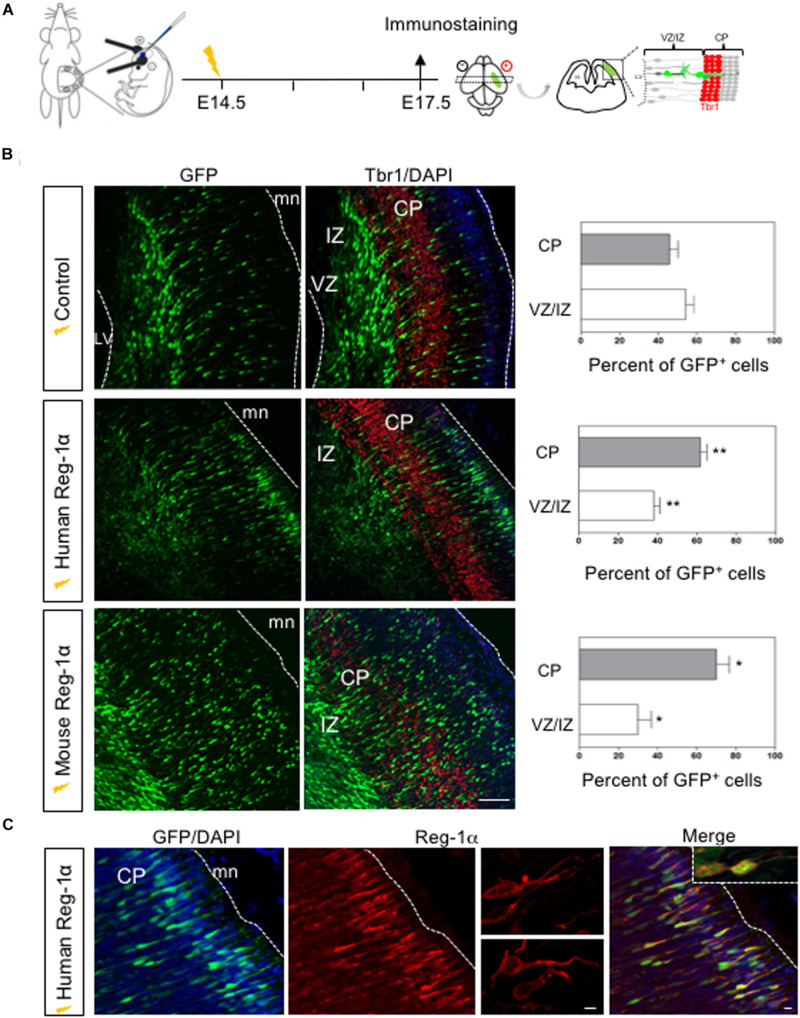
*In vivo* overexpression of Reg-1α induces a premature cortical migration. **(A)** Schematic representation of the experimental procedures used to electroporate *in utero* constructs encoding human or mouse Reg-1α cDNAs in E14.5 telencephalon. A GFP expressing plasmid was co-electroporated as tracer. Embryos were analyzed at E17.5. **(B)** Frontal sections of the telencephalon immunostained for GFP and Tbr1, a differentiation marker. Sections were counterstained with DAPI. The number of GFP+ cells present in the different layers were quantified and plotted against the total number of GFP+ present in the entire thickness of the section (graphs on the right). Bars represent the mean from a minimum of 3 independent embryos. Mann–Whitney test; *p < 0.05, **p < 0.01. Scale bar, 100 μm. **(C)** Representative confocal images of GFP+ cells in the CP after Reg-1α transfected neural cells. Sections were immunostained for Reg-1α. Note the presence of the protein on the neuronal surface. LV, lateral ventricle, CP, cortical plate, VZ/IZ, ventricular zone/intermediate zone. White lines indicate boundaries of the VZ or the meninges (mn). Scale bars, 10 μm.

We next asked if the effects of Reg-1α in NS were also occurring *in vivo*. To this end we overexpressed the human or mouse *Reg-1α* gene together with GFP (as tracer) in the cortex of E14.5 mouse embryos using *in utero* electroporation (Figure 6A). Three days after electroporation, brains were harvested, sectioned in the coronal plane and immunostained for Tbr1, an early post-mitotic neuronal marker, which is strongly expressed in the subplate at this developmental stage. In the developing telencephalon, progenitor cells that exit the cell cycle in the VZ migrate toward the cortical plate, providing an excellent model to study the effect Reg-1α on both proliferation and migration.

To determine the effect of Reg-1α, we divided the cortical neuroepithelium in two regions: the strongly Tbr1-positive area up to the cortical plate (CP), and the Tbr1-negative ventricular zone/intermediate zone (VZ/IZ; Figure 6B). In each one of them we compared the distribution of GFP-positive cells in control and Reg-1α electroporated embryos. In E17.5 control embryos, less than 50% of the GFP-positive cells had entered the CP (43.3 ± 3.7%; Figure 6B, gray bars) whereas the remaining GFP-cells were still found in the intermediate zone (56.6 ± 3.7%, white bars). In contrast, an increased fraction of progenitors electroporated with human Reg-1α were found in the CP (62 ± 3.1%, *p* < 0.0026; Figure 6B, gray bars) with a concomitant decrease in GFP-positive cells in the VZ/IZ (38 ± 3.1%, white bars). A similar effect was observed when cells were co-electroporated with mouse Reg-1α(mReg-1α) and GFP. A significantly higher percentage of mReg-1α–positive cells (70 ± 6.7%, *p* < 0.0137) were distributed throughout the CP with a decrease of cells in the VZ/IZ (29.9 ± 6.7%; Figure 6B) compared to control. Using GFP and Reg-1α labeling, we observed that Reg-1α localized mainly to the cell body and nerve processes of the electroporated cells (Figure 6C), which acquired a polarized and radially oriented morphology, typical of migrating cells in the developing cortical plate.

Altogether these data indicate that the overexpression of Reg-1α has an evolutionary conserved role in accelerating neuronal migration in the cortical plate.

### Reg-1α Regulates Cortical Neurogenesis

**FIGURE 7:**
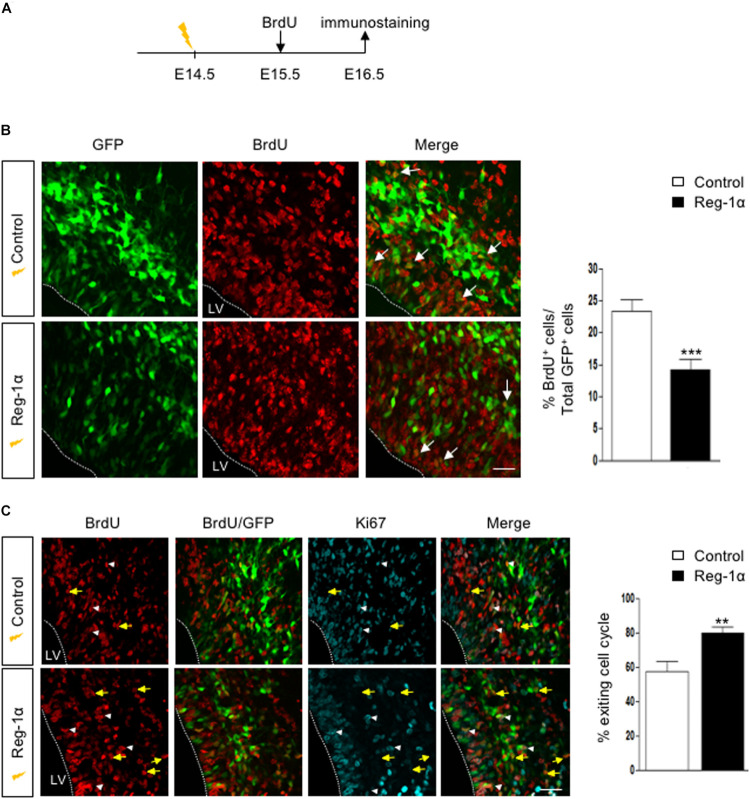
Reg-1α induces telencephalic progenitors to exit the cell cycle. **(A)** Timeline of human Reg-1α/GFP *in utero* electroporation and BrdU labeling of embryonic telencephalon. **(B)** Frontal sections of harvested brains immunostained for BrdU and GFP (arrows). The graph on the right shows the percentage of BrdU/GFP+ over the total number of GFP+ cells in the VZ/IZ. Reg-1α significantly decreases the number of BrdU+/GFP+ cellsScale bar, 50 μm. **(C)** Frontal sections of E16.5 embryos immunostained for BrdU and the cell-cycle marker Ki67. GFP/BrdU/Ki67 positive cells (white arrowheads) are actively cycling. GFP/BrdU positive cells (yellow arrows) have exited the cell cycle. The graph on the right shows the percentage of cells exiting the cell cycle, measured as the percentage of the GFP+BrdU+KI67- over the total number of GFP+BrdU+ cells. Bars represent the mean from a minimum of three independent embryos. Mann–Whitney test; **p < 0.01, ***p < 0.001. LV, lateral ventricle. Scale bar, 80 μm.

The Reg-1α-mediated increase of cell migration into the CP could be the result of the progenitors’ premature exit from the cell cycle associated with their differentiation. To test this possibility, we coupled electroporations of E14.5 embryos with subsequent BrdU injections at E15.5. Embryos harvested at E16.5 were immunostained for BrdU (Figure 7A). Reg-1α transfected cells were rarely BrdU-positive with a significantly lower percentage of co-labeled cells in the VZ (14.14 ± 1.7%, Figure 7B, bottom panel, arrows) when compared with controls (23.2 ± 1.8%, Figure 7B, top panel, arrows). To test if this reduction was associated with premature differentiation into neurons, we measured the cell cycle exit index by immunostaining for BrdU and Ki67, a cell proliferation marker (Figure 7C). GFP-positive cells labeled with both BrdU and Ki67 should reflect actively proliferating cells, whereas cells positive only for GFP and BrdU were considered as cells that already left the cell cycle. An increased proportion of cells electroporated with Reg-1α had left the cell cycle (Reg-1α : 79.7 ± 3.3% compared with GFP controls: 57.3 ± 5.7%), explaining the reduction of proliferating progenitors (Figure 7C). In agreement with an increased differentiation we also observed that at P0, Reg-1α overexpression increased the length of labeled callosal axons projecting from layer II/III cortical neurons (1058 ± 137 μm versus Gfp 707.7 ± 25 μm, *p* < 0.002, Mann–Whitney test; data not shown), further confirming the effect of Reg-1α observed *in vitro*.

Altogether our data demonstrate that overexpression of Reg-1α *in vivo* promotes cell cycle progression exit and promotes premature neuronal differentiation and migration to the upper layers further enhancing axonal elongation.

### Overexpression of Truncated Mutants Reg-1α in the Embryonic Mouse Neocortex

**FIGURE 8:**
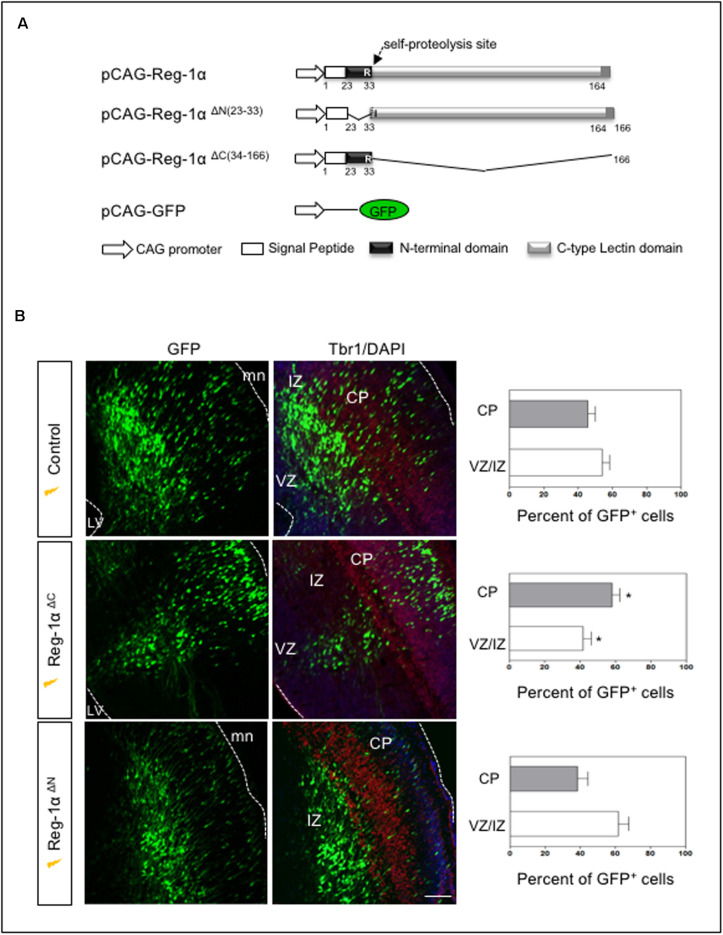
The N-terminal domain of Reg-1α mimics the effect of the full length protein. **(A)** Schematic representation of the different deleted versions of Reg-1α. **(B)** Confocal images of frontal sections from E17.5 embryos electroporated with GFP, and the deleted versions of Reg-1α as indicated in the panels. Sections were immunostained for GFP and Tbr1 and counterstained with DAPI. Quantification of GFP+ cells in the VZ/IZ and CP in the different conditions is plotted in the graphs on the right. Each bar represents the mean from at least three independent embryos. Mann–Whitney test (*p < 0.05, versus the control group). LV, lateral ventricle, CP, cortical plate, VZ/IZ, ventricular zone/intermediate zone. Dotted white lines indicate VZ or meninge (mn) boundaries. Scale bar, 100 μm.

**FIGURE 9:**
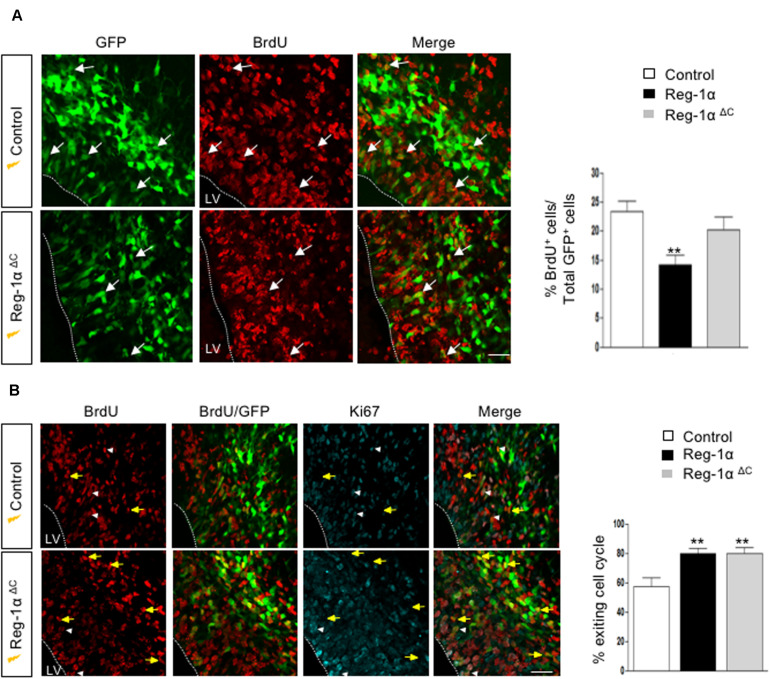
Reg-1α^Δ^ ^C^ promotes exit from the cell cycle. **(A)** Confocal images of frontal sections from E16.5 cortex electroporated at E14.5 with a Reg-1α^Δ^ ^C^ (*n* = 4) or Reg-1α (*n* = 3) and GFP (*n* = 5) plasmids. 24 h later, BrdU was injected intraperitoneally at E15.5. Sections were double labeled for BrdU/GFP. The graph shows the percentage of BrdU/GFP+ cells in the VZ/IZ. Scale bar, 50 μm. **(B)** Confocal images of frontal sections immunostained for GFP, BrdU and Ki67. Both Reg-1α and Reg-1α^Δ^ ^C^ increase the percentage of GFP + BrdU + KI67–/GFP + BrdU+. Bars represent the mean from 3 independent embryos. ANOVA followed by Dunnett’s multiple comparison test (**p < 0.01 versus the control group). LV, lateral ventricle. Scale bar, 80 μm.

Reg-α is cleaved into two domains (Nter and Cter). We thus asked whether the observed developmental activities of Reg-α required the participation of both domains or one of them was sufficient to mimic the effect of the entire protein. To address this question, we generated two mutant versions of human Reg-1α in the pCAG expression plasmid: Reg-1α^ΔN (23–33)^, which lacks the N-terminal domain and Reg-1α^ΔC (34–166)^, lacking the C-type lectin domain (Figure 8A). We then overexpressed the resulting human mutated forms by means of *in utero* electroporation following the same protocol we used for the intact protein (Figure 6). Comparison of the distribution of GFP-positive cells across the thickness of the cortical neuroepithelium showed that Reg-1α^ΔN^ did not promote progenitor migration. Rather, GFP-positive cells were found preferentially in the VZ/IZ (Reg-1α^ΔN^: 61.7 ± 6.1%) compared with GFP controls (56.6 ± 3.7%; Figure 8B) and accordingly a smaller proportion of cells ended up in the CP (Reg-1α^ΔN^ 38.4 ± 6%) as compared to GFP controls (43.3 ± 3.7%). In contrast, Reg-1α^ΔC^ was sufficient to promote the accumulation of larger fraction of GFP-positive cells in the CP (58.1 ± 4.5%, *p* < 0.0317, Figure 8B, gray bars) with a consequent decrease of GFP-positive cells in the VZ/IZ (Reg-1α^ΔC^: 41.9 ± 4.5%, white bars). Similarly to what observed for the intact protein, Reg-1α^ΔC^ promoted a premature exit of progenitor cells from the cell cycle. Reg-1α^ΔC^ caused a slight but not significant reduction of BrdU/GFP-positive cells in the ventricular zone compared to control (Figure 9A) but measure of the index for cell cycle exit highlighted a significant increase (Reg-1α^ΔC^: 80.04 ± 3.8%, comparable to that observed with Reg-1α (Reg-1α : 79.7 ± 3.3% compared with controls (57.3 ± 5.7%, Figure 9B).

Thus, Reg-1α^ΔC^ alone can mimic Reg-1α activity in the cortical plate, whereas the C-terminal domain has no effect, demonstrating a critical role for the N-terminal undecapeptide domain of Reg-1α in neuronal differentiation.

## Discussion

Different members of the Reg family of C-type lectin proteins are expressed in nervous system as for example in the developing and regenerating rat motor and sensory neurons (Namikawa et al., 2005; Parikh et al., 2012), in central neurons after traumatic brain injury (März-Weiss et al., 2011) or as consequence of excitotoxic brain injury (Haldipur et al., 2014). Among the different family members, Reg-1α has been shown to be expressed during embryonic development and protein levels are stage-dependent while mRNA levels are stable. Very recently, it has been shown that microRNA-7 directly targets the murine *Reg1* modulating its expression in pancreatic cells (Downing et al., 2019). miR-7 is expressed in various regions of the brain of humans and mice (Farh et al., 2005; Landgraf et al., 2007) and could be affecting the Reg-1α expression in this context. We previously established that Reg-1α promotes neurite outgrowth from cultured hippocampal neurons (Acquatella-Tran Van Ba et al., 2012). In the present study, we demonstrated a novel role for Reg-1α in promoting migration and differentiation of telencephalic progenitor cells. This function is mediated by its N-terminal domain, which mimics the effect of the intact protein and involves the activation of GSK-3β.

Reg-1α induces mouse embryonic stem cells (mESCs) to acquire an endoderm-like phenotype (Jing et al., 2010) and restrains proliferation of gastrointestinal cell types (for review Chen et al., 2019) suggesting a possible general role of this protein in cell differentiation. Our study supports this idea. We showed that both Reg-1α and its receptor Extl3 are expressed during cortical development in progenitors, post-mitotic NeuN-positive neurons and Calbindin interneurons. Reg-1α overexpression in neural progenitors induces their premature exit from the cell cycle and promotes their differentiation increasing the number of cells present in the CP. A similar effect is observed in neurosphere cultures derived from embryonic telencephalon, in which Reg-1α promotes progenitors’ differentiation. Notably, Reg-1α does not push cells out of the cell cycle but also increases the elongation of their neurites and their migration out of the neurospheres. All of these effects are mediate by extracellular Reg-1α because addition of anti-Reg-1α antibodies in the cell culture medium are sufficient to reduce these effects, well in line with previous studies showing that specifically promotes axon elongation of cultured hippocampal neurons (Acquatella-Tran Van Ba et al., 2012). Reg-1α might also have opposite roles. For example in the digestive system exogenous Reg-1α stimulates the PI(3)K/ATF-2/cyclin D1 signaling pathway, thereby inducing β-cell regeneration. Furthermore, when applied exogenously to the culture media of cells that express EXTL3, Reg-1α induces cells proliferation via MAPK and cyclin D1, with concomitant induction of MAPK phosphatase-1 (MKP-1). Conversely, when Reg-1α overexpressed within cells or added at very high concentration, it inhibits growth likely via a binding to and inactivation of MKP-1 (Mueller et al., 2008). Inhibition of cell growth may lead to transdifferentiation to other cell type of gastrointestinal tract (Miyaoka et al., 2004; Mueller et al., 2008). In analogy with our *in vivo* approach, MPK-1 could represent a potential intracellular partner of Reg-1α overexpressed that could stop neural progenitor division and modify their terminal differentiation program. Nevertheless, we have shown that GSK-3β, a key component of several transduction cascades, may mediate Reg-1α activity. GSK-3β is an important hub of different signaling cascades and its activity is involved in differentiation and migration of embryonic/adult neural stem cells and differentiation of cancer cells with stem cell-like characteristics, such as glioblastoma cells (Korur et al., 2009; Manceur et al., 2011; Lee et al., 2013).

GSK-3β is also central to the Wnt/β catenin signaling pathway, which plays a key role in neural precursor self-renewal, laminar fate determination and radial migration of pyramidal precursors. This suggests a relationship between Reg-1α and the Wnt signaling pathway during cortical development. Accordingly we show that Reg-1α induces the inhibition of GSK-3β activity (indicated by p^Y216^GSK-3β decrease and p^Ser9^GSK-3β increase) and the increased of content of cytoplasmic β-catenin and its nuclear translocation (Supplementary Figure 3). A similar relationship between Reg-1α and Wnt signaling has been suggested in human liver tumors, in which Reg-1α upregulation is associated with mutations in the β-catenin gene (Cavard et al., 2006). Furthermore, activation of the β-catenin pathway in mESCs has been linked to the specific up-regulation and secretion of Reg-1α. Notably, in cortical neurons that express Tau P301L and thus present alterations in the microtubule network, Reg-1α induces GSK-3β activation (Moussaed et al., 2018). Nevertheless, the link between Reg-1α and Wnt signaling *in vivo* needs to be further clarified.

Reg-1α is highly susceptible to proteolysis (Cerini et al., 1999) and its cleavage generates two fragments. Our study unveils that the undecapeptide N-terminal peptide, which is physiologically generated by trypsin cleavage at the Arg^11^-Ile^12^ position, is necessary and sufficient to mimic Reg-1α functions during cortical development. This peptide is also responsible of inducing neurite outgrowth from cultured embryonic hippocampal neurons (Supplementary Figure 4). The function of the 11 amino-acids N-terminal peptide does not seem to be shared by other Reg family members, such as RegIIIγ, RegIIIβ (Konishi et al., 2013), thus representing a unique characteristic of Reg-1α. Previous studies have, however, shown that the truncated N-terminus of PAPIII serves as scaffold for neurites and promote neurite outgrowth (Konishi et al., 2013). This domain (called ΔN-PAP-III) can form fibrils that, when used as a substrate, promote neuritic elongation. The report contrasting with our results could be due to the differences in the experimental set up: while Konishi et al. (2013) used the PAP-III fibrils (obtained from recombinant protein) as a coated matrix for neuronal support, we overexpressed Reg-1α and its domains in neurons. Moreover, Reg-1α and Reg-IIIγ are two distant family members and sequence comparison of their N-terminal peptides shows a very limited identity of 27% (with the human sequence of Reg-1α), which could also explain this apparent discrepancy. In another study on HIP/PAP human protein (RegIIIβ), the COOH terminal domain of the molecule was reported to bind bacterial peptidoglycan allowing bactericide function which is prevented by the presence of the N-terminal segment (Mukherjee et al., 2009).

## Conclusion

In conclusion, we proposed that the migration and the differentiation of neural progenitors are induced by Reg-1α via the regulation of the downstream GSK3-β signaling. These functions are mediated by N-terminal segment of Reg-1α so that, Reg-1α through its N-terminal undecapeptide represents a potentially important factor involved in cortical neurogenesis.

## Data Availability Statement

The datasets presented in this study can be found in online repositories. The names of the repository/repositories and accession number(s) can be found in the article/ Supplementary Material.

## Ethics Statement

The animal study was reviewed and approved by all animal procedures were performed in strict adherence to the European Union directive 2010/63 and the ARRIVE guidelines upon CEEA-LR-12031 authorization.

## Author Contributions

IA-T, AM, and FT designed the research. MV, FN-L, MR, and FT performed the *in utero electroporation* studies. IA-T and AM performed the neurosphere culture and FACS analysis. MS performed the qPCR studies. MM and M-CL performed the immunoblot studies. IA-T, MV, AM, and FT analyzed the data and wrote the manuscript. PB and J-MV revised the manuscript. All the authors read and approved the submitted version.

## Conflict of Interest

The authors declare that the research was conducted in the absence of any commercial or financial relationships that could be construed as a potential conflict of interest.
