# Supplementary figures and images for: Reg-1α Promotes Differentiation of Cortical Progenitors via Its N-Terminal Active Domain
Source: Front Cell Dev Biol. 2020 Aug 13;8:681. doi: 10.3389/fcell.2020.00681 (PMC7443566; doi:10.3389/fcell.2020.00681)

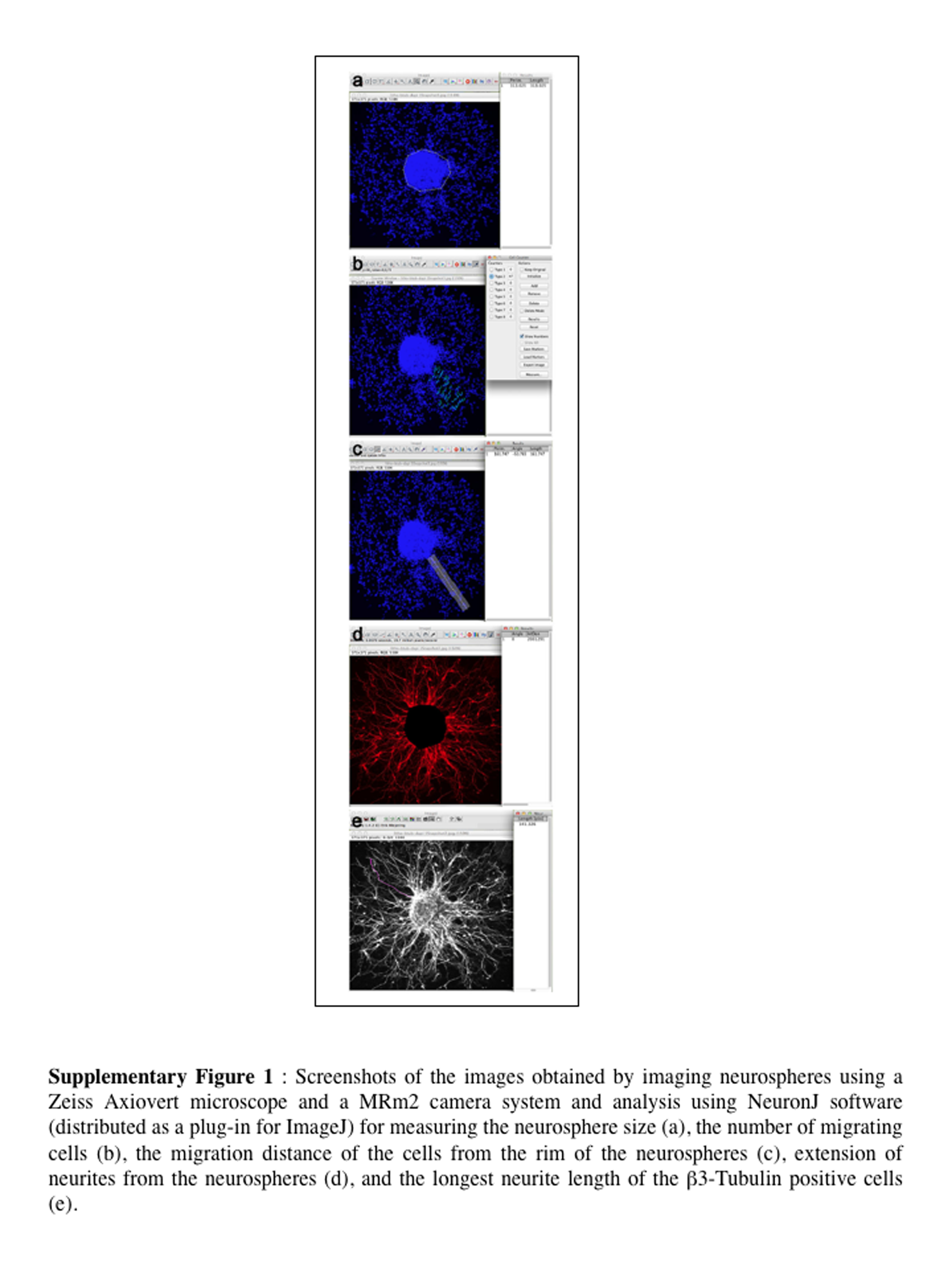

Supplement: Supplementary file 1 [file Image_1.TIF]

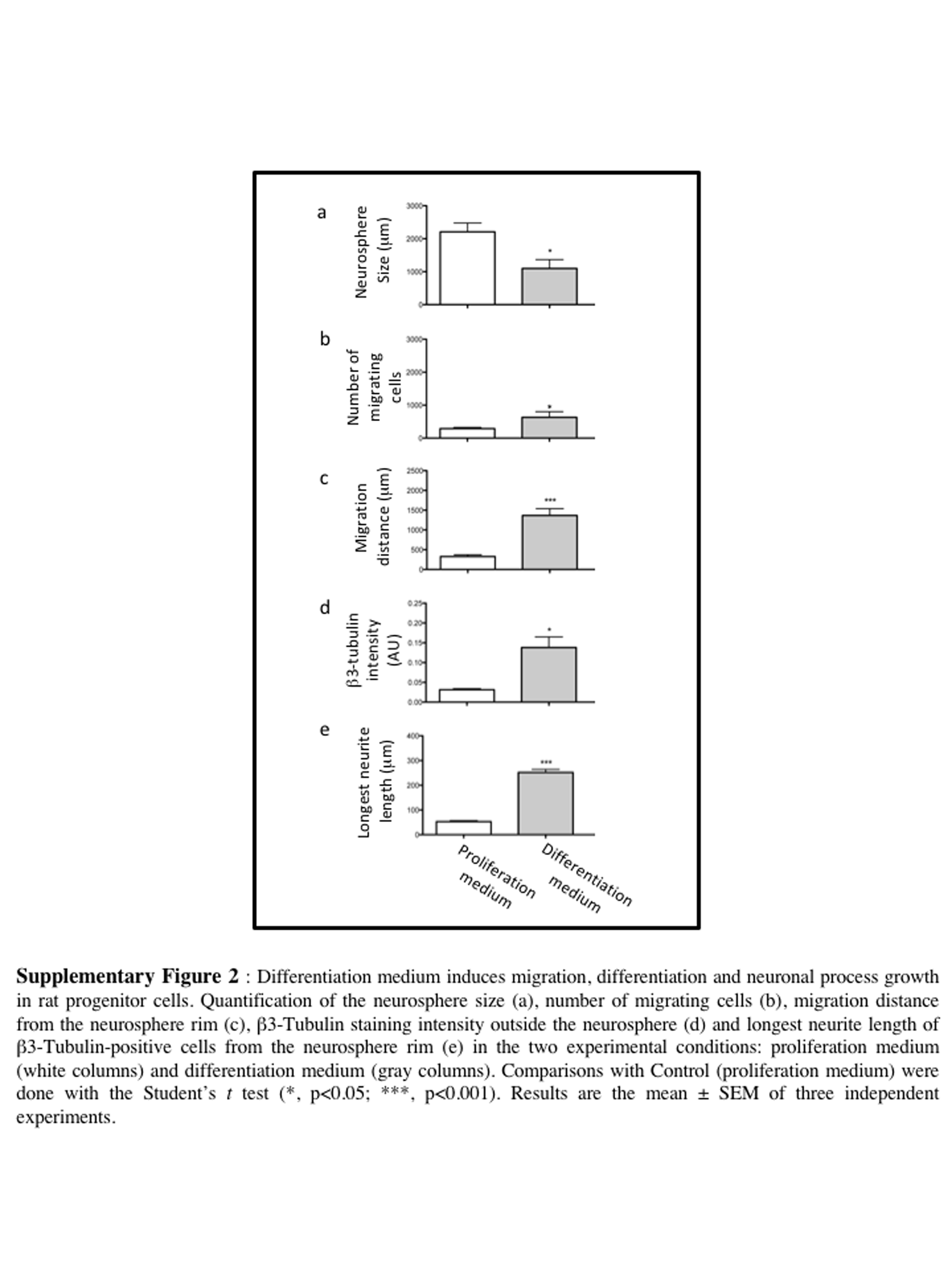

Supplement: Supplementary file 2 [file Image_2.TIF]

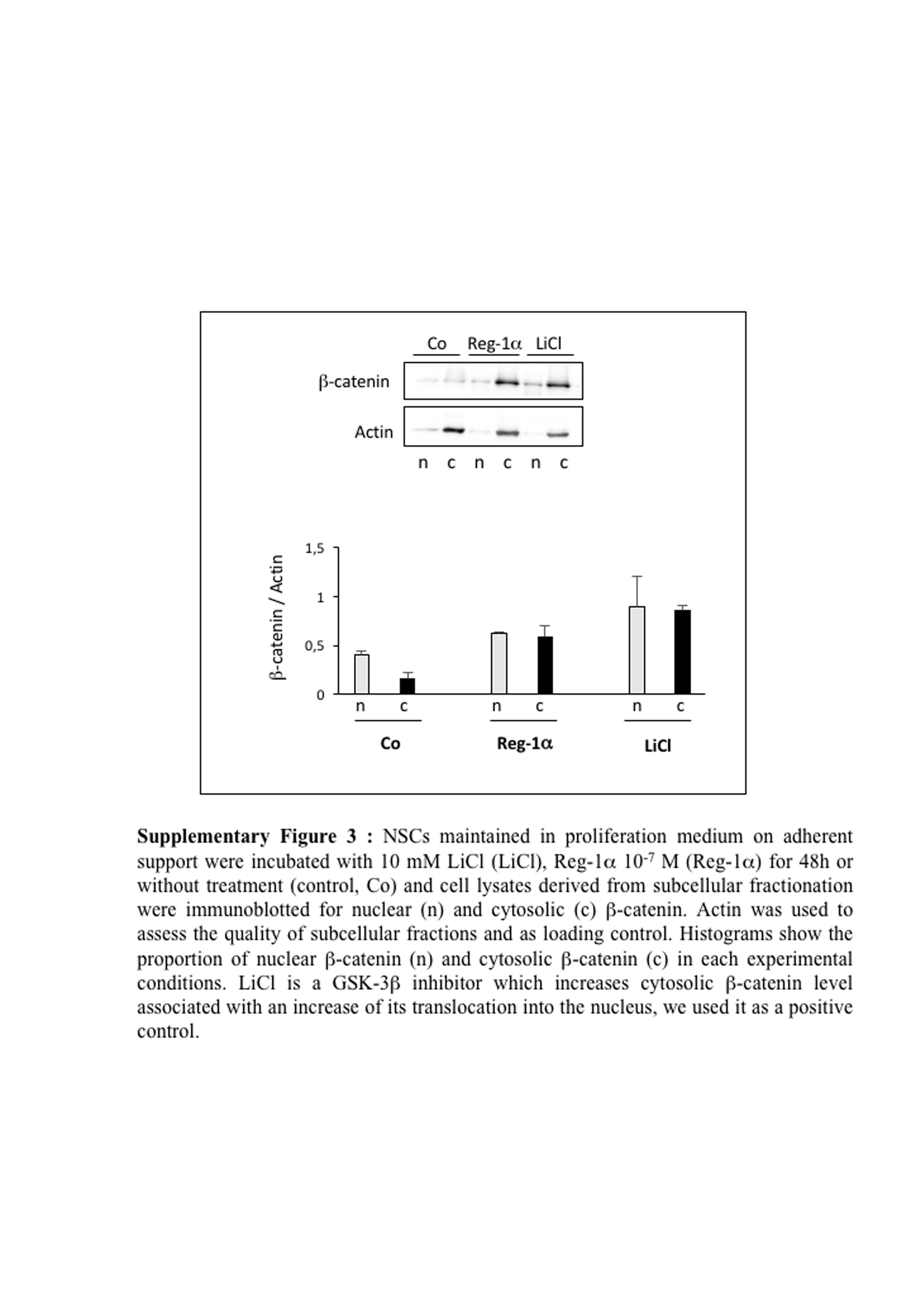

Supplement: Supplementary file 3 [file Image_3.TIF]

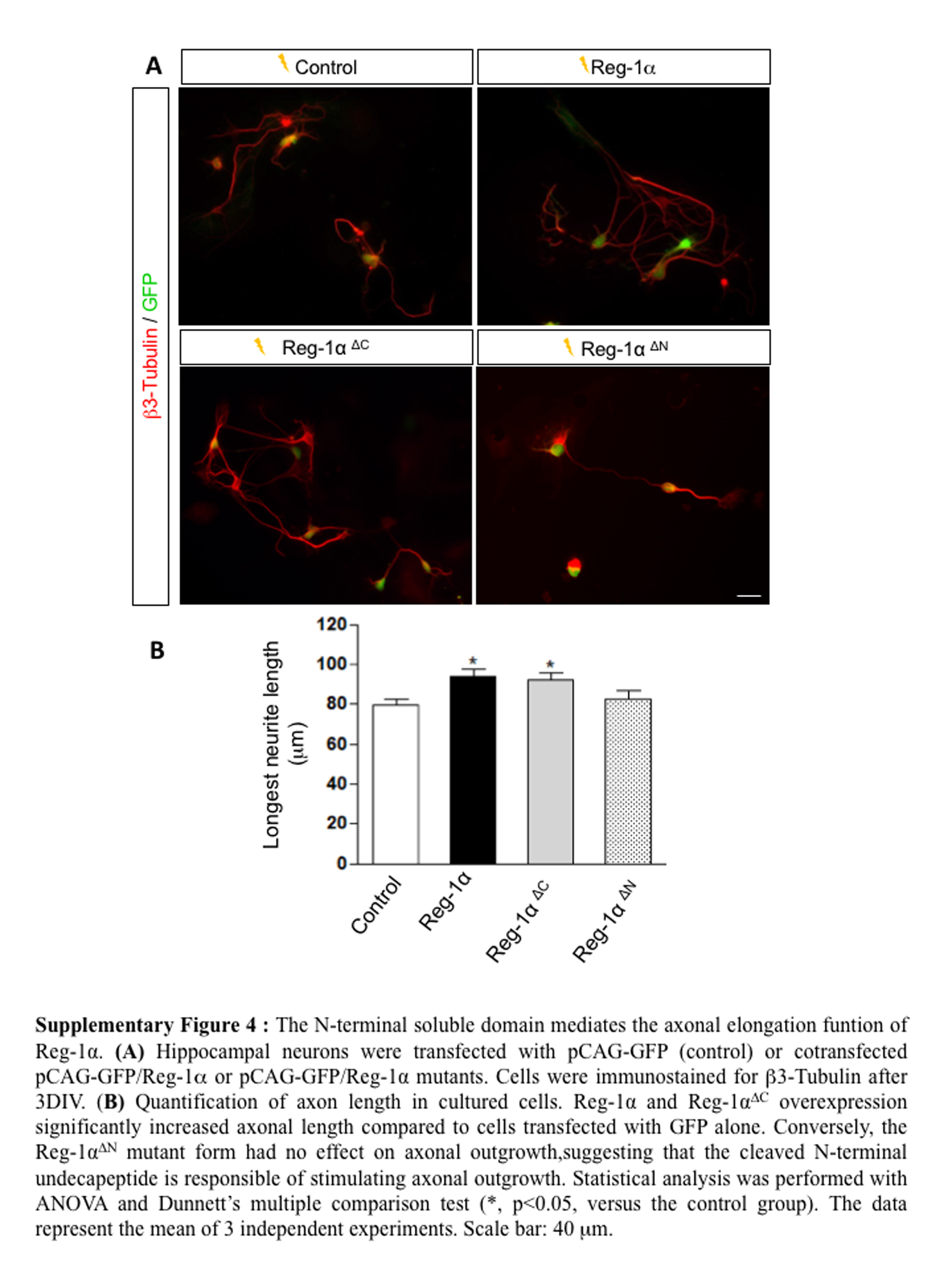

Supplement: Supplementary file 4 [file Image_4.TIF]
